# Traumatic tension pneumocephalus: a case report and perspective from Indonesia

**DOI:** 10.3389/fneur.2024.1391270

**Published:** 2024-05-03

**Authors:** Goran Latif Omer, Riccardo Maurizi, Gianluca Velletrani, Beatrice Francavilla, Sahand S. Ali, Aland Salih Abdullah, Stefano Di Girolamo

**Affiliations:** ^1^Department of Clinical Sciences, University of Sulaimani-College of Medicine, Sulaymaniyah, Iraq; ^2^Department of Otorhinolaryngology, University of Rome Tor Vergata, Rome, Italy; ^3^Department of Basic Medical Sciences, University of Sulaimani-College of Medicine, Sulaymaniyah, Iraq

**Keywords:** tension pneumocephalus, endoscopic endonasal, head trauma, craniotomy, ethmoidal defect

## 1 Introduction

We read with great interest the recent Article by Harlyjoy et al. (1) focusing on the management of traumatic tension pneumocephalus (TP) and the associated challenges in treating patients with head trauma in low- and middle-income countries (LMICs) (1). The authors' exploration into optimizing treatment protocols to reduce hospitalization times and minimize post-operative complications is both timely and pertinent. They notably underscore the critical need to address delays in the management of traumatic brain injuries by enhancing the utilization of health resources. We would like to provide our insights based on our experience in managing TP caused by post-traumatic ethmoidal damage, further contributing to this essential dialogue.

## 2 Discussion

While Pneumocephalus is a condition characterized by the presence of air in the intra cranial space caused by a breach in the cranium dural barrier, in TP air is progressively accumulated in the intra cranial space by a “ball valve” or “inverted pop bottle” mechanism. This mechanism categorizes TP as emergency condition due to the compressive effect of the trapped air on the brain. In the management of TP, the primary objective is the emergent decompression to alleviate intracranial pressure, combined with the repair of the causative defect (2). Endoscopic multilayer repair is the standard treatment for closing the craniodural breach. To achieve adequate intracranial decompression, the historical craniectomy has been replaced with the more recent craniotomy (3). Despite its efficacy, craniotomy is associated with a notable risk profile including soft tissue infection, extradural abscesses, subdural empyema, bone flap infection, and postoperative intracranial infection (4). Shi et al. (5) reported their experience with post craniotomy intracranial infection (PCII), showing a PCII rate of 6.8% among 5,732 patients.

Recent studies have explored various endoscopic techniques that simultaneously address the resolution of traumatic tension pneumocephalus (TP) and the closure of the bone defect responsible for cerebrospinal fluid leakage in a single surgical stage (6, 7). The development of new techniques and more angulated instruments for endoscopic surgery has increased the possibility of accessing more challenging endonasal areas (8). Single-step endonasal procedures may effectively treat both the TP and the craniodural defect concurrently, significantly lowering the risks of complications and shortening the duration of hospital stays. Patients treated with these strategies can expect reduced postoperative morbidity, quicker discharge, and a faster return to work. This is particularly relevant in LMICs, where the healthcare infrastructure may not support extensive postoperative care, and where the economic impact of prolonged hospitalization can be substantial.

To the best of our knowledge, the literature offers only four examples of TP resulting from post-traumatic ethmoidal roof damage, complicating the development of precise management guidelines. However, with equal effectiveness, the more appropriate direction should focus on procedures that allow a reduction in post-operative hospital stays and complications rate.

In conclusion, the adoption of endonasal endoscopic approaches for the first-line treatment of symptomatic TP due to post-traumatic ethmoidal defects offers a promising opportunity to improve patient outcomes not just in LMICs, but globally. This method underscores the potential of minimally invasive surgical techniques in TP management, promoting a model that prioritizes efficiency, safety, and accessibility while addressing the challenges of limited healthcare infrastructure and resources.

## Author contributions

GLO: Writing – review & editing. RM: Writing – original draft, Writing – review & editing. GV: Writing – review & editing. BF: Writing – review & editing. SSA: Writing – review & editing. ASA: Writing – review & editing. SD: Writing – review & editing.
